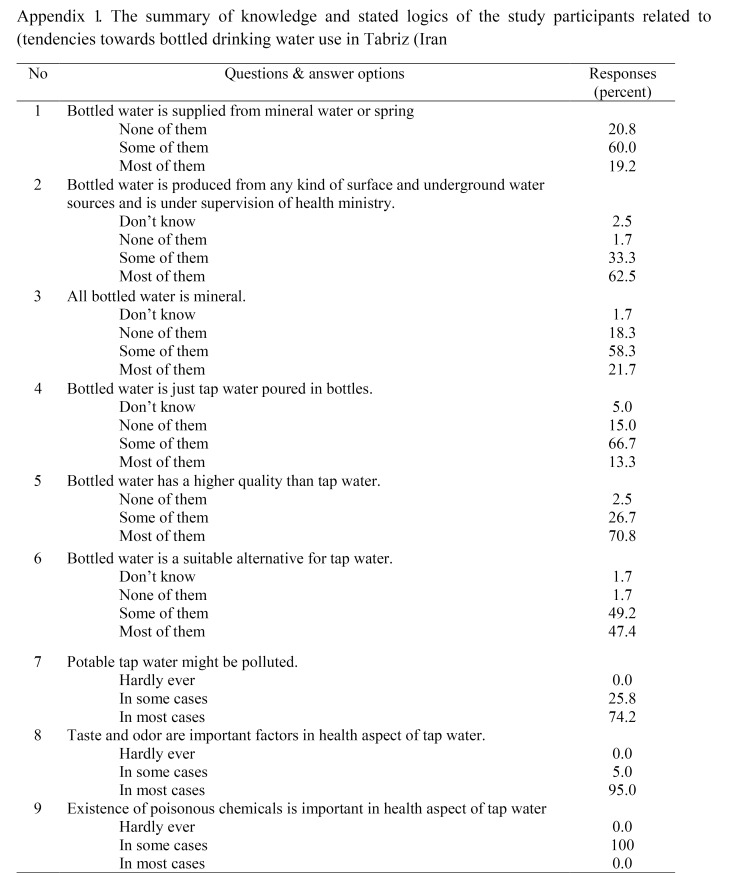# Tendencies towards bottled drinking water consumption: Challenges ahead of polyethylene terephthalate (PET) waste management

**DOI:** 10.34172/hpp.2021.09

**Published:** 2021-02-07

**Authors:** Hassan Aslani, Parisa Pashmtab, Abdolreza Shaghaghi, Asghar Mohammadpoorasl, Hassan Taghipour, Mahsa Zarei

**Affiliations:** ^1^Health and Environment Research Center, Tabriz University of Medical Sciences, Tabriz, Iran; ^2^Department of Environmental Health Engineering, Tabriz University of Medical Sciences, Tabriz, Iran; ^3^Department of Health Education and Promotion, Tabriz University of Medical Sciences, Tabriz, Iran; ^4^Department of Statistics and Epidemiology, Tabriz University of Medical Sciences, Tabriz, Iran; ^5^Faculty of Civil Engineering, University of Tabriz, Tabriz, Iran

**Keywords:** Bottled water, Waste bottle, Management, Tendency, Iran

## Abstract

**Background:** The main objective of this study was to investigate the logics behind tendencies towards bottled drinking water usage in spite of availability of treated tap water. The amount of waste bottle is also estimated in Iran and managing principles for resolving the issue presented.

**Methods:** A questionnaire was used to survey the logics behind tendencies toward bottled drinking water consumption among 120 participants. In order to estimate the quantities of the PET wastes produced in the country, data about bottled water production rate as well as volume of the imported and exported drinking bottled water were collected from 1962 to 2015 and applied in the calculations.

**Results:** Findings suggested that about 0.026 to 3.86 billion liters (about 1.04 billion literson average) of bottled water was consumed annually between 2000 and 2015. Furthermore, bottled water consumption increased from 0.41 to 48.9 L/capita-year within the same time period. In the meantime, the plastic bottle waste generation rate rose from 12.84 to 1519.38 g/capita-year. There is no efficient and suitable system for managing and recycling waste bottles in the country. The perceived unreliability of tap water quality was the main reason of bottled water consumption among 74% of the respondents.

**Conclusion:** To reduce bottled water consumption and the associated harmful environmental and health consequences, measures such as informing people, validating public water supply quality, preventive rules enactment, and establishing extended producer responsibility (EPR) are highly recommended.

## Introduction

In spite of improved quality of drinking water in water distribution systems, the use of bottled water is progressively growing.^1-3^ Bottled water consumption is increasing annually by 7% on average worldwide.^4^ Global consumption of bottled water was about 232 billion liters (61.4 billion gallons) in 2011, and estimated to increase to 513 billion liters by 2025.^5^ North America has the highest rate of bottled water consumption (30%) followed by Europe with 29% and Asia with 27%, and 14% in other parts of the world.^6^ It has been reported that during the last two decades bottled water consumption, from non-existent in the early 1990s to the second biggest market in the beverage industry, has increased dramatically.^7^ According to a recent report, global bottled water market valued as $US198 billion in 2017. It has been recently reported that a million plastic bottles are sold every minute around the world, and this figure may increase another 20% by 2021.^8^ Based on this report more than 480 billion plastic drinking bottled were sold in 2016 across the world, which was almost 60% higher in comparison with a decade ago. According to the most up to date data presented by Euro monitor International’s in global packaging trends report, this figure will increase to 583 billion by 2021.

Some studies tried to address why people choose to pay for a good (bottled water), that is sufficiently provided and available to them in a very lower cost. Results indicated that some people, especially those living in locations where tap water quality standards have been violated, believe that the bottled water is safer or tastes better.^9^Another study suggests that consumers perceive bottled water as a pure and healthy substitute to tap water and link it to their health status.^10^ A study in France investigated historical increase in bottled water consumption. The results indicated that purity, nature, and health were reasons consumers stated for their tendency on bottled water. However, there are limited number of academic studies exists to address why people pay for bottled water.

Bottled water use can be controversial from two prospects: initially, the packed water quality and its corresponding health consequences; secondly, the high energy demand for its production and distribution.^9,10^ Meanwhile, the management of waste generated by discarded bottles is of great concern from an environmental health point of view.^11^ The most common plastic in use to produce containers of packed drinking water is polyethylene terephthalate (PET), which takes 400 years to naturally decompose in the nature. While this plastics are highly recyclable, less than half the bottles sold collected for recycling and just 7% of them are being recycled. Due to its low cost, easy transportation, low weight, and high resistance as well as lack of taste and aesthetic problems, PET is widely used for packaging a wide variety of edible products including water. As plastic bottles are not readily biodegradable, the main challenge is their durability in the environment.^12^ The discarded PET bottles have a high potential to be an environmental catastrophe (especially in economically developing countries) if not recycled sufficiently. They end up in unsanitary landfills or dumpsites and in turn contribute to contamination of soil, water, oceans, seas, coastal areas, and even air pollution.^3,13^ When bottles are dumped in landfill sites, they occupy huge space. In addition, since bottled water manufacturers mainly rely on fossil fuels, energy consumption is another major issue in production and distribution of bottled water. The amount of energy required for treating water, producing plastic bottles, and transporting bottled water to shopping centers can further aggravate the conditions. The bottled water packaging, their labeling, and cooling at purchase sites are other sources of energy consumption in bottled water manufacturing. In short distance transportations, more than 70% of the spent energy is related to plastic bottle production, while in long distances, most of the required energy is expended on the transportation.^14^ Based on empirical research evidence, the amount of total energy required for production of 1 liter of bottled water has been estimated to be about 5.6-10.2 MJ/L^15^ in comparison with 0.005 MJ for treatment and distribution of tap water.^16^ The environmental effect of consumption of bottled water is about 100 times more than that of potable tap water.^17^ Beside all environmental consequences, it is forecasted that production of each bottle consumes three times as much water than required to fill the bottle (7 -sustainability).

Bottled drinking water supplies are essential in regions without access to treated pure drinking water. However, their use could be paradoxical in countries such as Iran with almost easy and widespread access to treated tap water across urban areas, on the one hand and limited natural resources for production of drinking water and environmental effects of generated waste of bottled water on the other. Accordingly, this study aimed to examine the logics behind the tendencies towards bottled drinking water usage, while potable drinking water with a standard quality is available. Furthermore, as there is lack of information about bottled water waste in Iran and almost all around the world, estimating the amount of waste generated from these bottles and presenting managing principles to resolve the issue have been the other objectives followed in this work.

## Materials and Methods

### Tendencies towards bottled drinking water use

By considering the results of a similar study in Mashhad, Iran,^18^ sample size was estimated using Cochran formula with confidence level equal to 95%, precision of 0.1, and *P* equal to 0.25. The minimum sample size was estimated to be 72 people, which by considering 25% for missing data increased to 120. Then convenience sampling method was used to recruit 120 study participants as users of bottled drinking water from small retailers and mega malls in Tabriz, the capital city of East Azerbaijan province. A researcher-designed questionnaire was developed to collect data about the demographic characteristics of the study attendees (e.g. age, sex, educational level, occupation, approximated income level, and place of residence) as well as their rationales to use bottled drinking water (32 questions). The reliability (test-retest in 16 participants; minimum Spearman’s correlation coefficient equal to 0.78) and validity (content validity by using 8 experts viewpoints) of the designed questionnaire were verified before its application. The participants were divided into two educational groups based on their academic degrees. The participants with master’s and Ph.D. degrees were classified as highly educated, while those with bachelor’s degree or lower were classified as middle educated. For data analysis, SPSS Statistics for Windows version 21.0 (IBM Inc., Armonk, NY, USA) software was used implementing one-way analysis of variance and Chi-square tests.

### Estimation of the PET wastes generated from the bottled drinking water consumption

In order to estimate the quantities of the produced PET wastes resulting from bottled drinking water use in Iran, data on production rate as well as imported and exported drinking bottled water were collected, between 1962 and 2015, from relevant organizations such as the Iranian National Statistics Organization, Customs Administration, and Ministry of Industry, Mine and Trade.

In the next stage, unit conversion was performed preferably from liter and metric tons of consumed water to kg and tons of generated PET wastes. Most of the bottled water production companies are not working with their full capacity due to issues such as economic problems, market demand, and higher production costs. Therefore, in order to have a reliable estimation, after consultation with informants, 40% of the nominal capacity of the bottled water producer companies was considered for calculating the magnitude of PET wastes. Accordingly, the total amount of bottled water consumed in the country was calculated based on equation 1. The per capita PET waste production was calculated considering the country’s population for each year and types of PET bottles in use for drinking water packaging (0.5 L and 1.5 L). According to the information provided by the producers of bottled drinking water and also available data from the Iranian Ministry of Industry, Mine and Trade, on average, 60% and 40% of bottled waters are supplied in 0.5 L and 1.5 L bottles, respectively.^19^ Accordingly, equations 2 and 3 were used to calculate the numbers of the 0.5 L and 1.5 L produced bottled water, correspondingly.

The quantity of PET water bottles was converted to weight (tons) for the purpose of this study. Also, through weighing the container of ten common types of bottled water in Iran by research team, the average weight of each 0.5 L and 1.5 L bottle was determined as 19 g and 31 g, respectively, so in our calculation we assumed these weights as representative for these bottles in Iran. However, as bottle manufacturing techniques and machines available varying around the world, these numbers can be adjusted in each country. The associated PET wastes introduced from both 0.5 L and 1.5 L bottles to either solid waste stream or environment were calculated employing equations 4 and 5.

Eq. 1 $$Total\;produced\;bottled\;water\;\left[\frac{tons}{year}\right]=\;(produced\;bottled\;water\;+\;imported\;bottled\;water)\;-\;\exp orted\;bottled\;water$$

Eq. 2 $$Number\;of\;the\;0.5\;L\;bottled\;water\;(10^{6})=\;\frac{\left(Total\;production\;of\;the\;bottled\;water\;(ton\;year^{-1})\times 0.6\times 1000(L\;ton^{-1})\right)}{0.5\times 10^{6}}$$

Eq. 3 $$Number\;of\;the\;1.5\;L\;bottled\;water\;(10^{6\;})=\;\frac{\left(Total\;production\;of\;the\;bottled\;water\;(ton\;year^{-1})\times 0.4\times 1000(L\;ton^{-1})\right)}{1.5\times 10^{6}}$$

Eq. 4 $$Total\;weight\;of\;the\;PET\;wastes\;(tons)\;from\;the\;0.5\;L\;bottles=\frac{N\;(Millions)\times 19(gr)\times 10^{6}\;(conversion\;factor)}{1000(g\;kg^{-1})\times 1000(kg\;ton^{-1})}$$

Eq. 5 $$Total\;weight\;of\;the\;PET\;wastes(tons)\;from\;the\;1.5\;L\;bottles=\frac{N(Millions)\times 31(g)\times 10^{6}(conversion\;factor)}{1000(g\;kg^{-1})\times 1000(kg\;ton^{-1})}$$

The sum of the mass of 0.5 L and 1.5 L PET wastes calculated by equations 4 and 5 was considered as the total PET waste generated from bottled drinking water use.

Based on the estimated data on the volume of PET waste generation from bottled drinking water use between 1962 and 2015, the annual average of the PET wastes associated with bottled drinking water and also per capita PET waste generation rate for the upcoming twelve years i.e. 2016-2028 was estimated using an artificial neural network.

### Review of the current administrative procedures for PET wastes in Tabriz city 

The required data about management procedures for PET wastes associated with the bottled drinking water were collected through completing a checklist, visiting and observing the central waste disposal site, and interviewing relevant authorities.

## Results

### Demographic and behavioral characteristics

The study participants consisted of 56 females and 64 males with the mean age of 37 (SD: 9.3), 83% of whom had an academic degree. According to the study results, 99.2% of the interviewees almost always consumed bottled drinking water. Notably, about 82.7% of the respondents, ordinary people, showed an insufficient level of knowledge about the scientific definition of bottled drinking water or the source of its production. The stated rationales of the subjects behind the use of bottled drinking water in the presence of treated tap water are represented in Table S1 (Appendix 1).

### Consumption of bottled drinking water and PET wastes produced

Table 1 presents the amounts of the estimated produced bottled drinking water (tons and billions of liters) in Iran and the imported and exported quantities throughout the 53-year time period from 1962 to 2015. The total (tons) and per capita (grams) amounts of the estimated PET wastes associated with bottled drinking water consumption during the time period are also reported in this Table. According to Table 1, in 1962 only about 0.0017 billion liters of bottled water were consumed, and 54.93 tons of related PET wastes were generated in Iran.

### Per capita use of bottled drinking water

**Figure 1:**
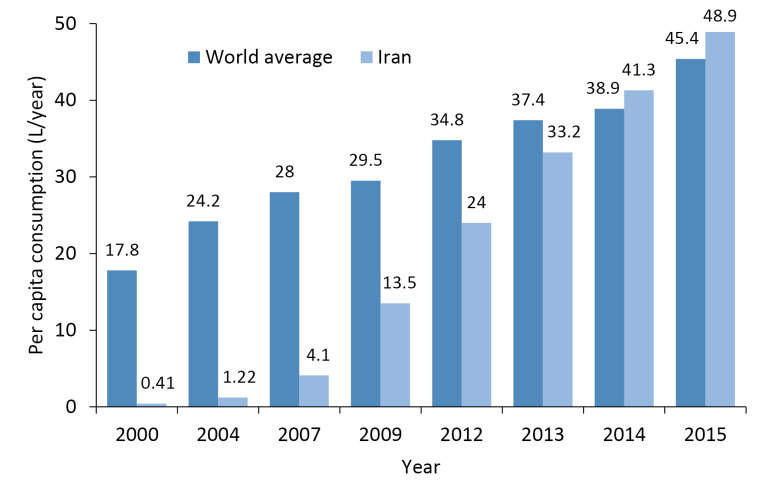
Comparison of annual per capita use of bottled drinking water in Iran and the world, between 2000 and 2015.

Figure 1 shows the trend of changes in average per capita bottled water in Iran and the average global, during fifteen years (2000-2015).

**Figure 2:**
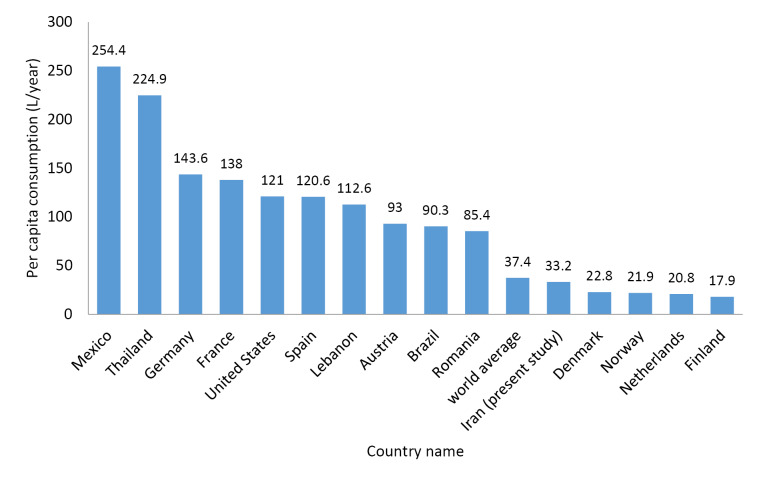
Comparison of annual per capita use of bottled water in Iran with the world average, and other countries in 2013.

The per capita consumption level of the bottled drinking water (L year^-1^) in Iran has been compared with that of other countries in Figure 2. As can be seen, per capita consumption in various countries is different, and per capita consumption in Iran is nearly like the global average.

### Forecast of the amount of PET waste generation

**Figure 3:**
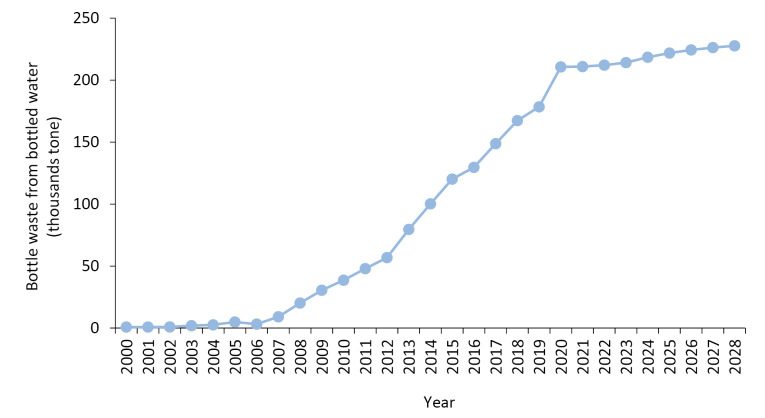
Estimated PET bottle wastes in Iran between 2000 and 2028.

To estimate the amount of PET bottled water waste generation between 2016 and 2028, artificial neural network model was used. The trend of waste bottle generation between 2000 and 2028 is displayed in Figure 3. It can be seen that waste generation from discarded bottles will grow by 2028.

## Discussion

According to Table S1 about 74% of the participants believed that the tap water might be contaminated frequently and cannot be a reliable source from the quality aspect. The findings also revealed that concerns over contamination of the tap water by poisonous chemicals (100%), microbial agents (98.3%), nitrate compounds (96.7%), undesirable hardness and taste (95.8%), and disgusting odor of the tap water (95%) were major justifications to select bottled drinking water instead of tap water use. Water treatment procedures in the studied city (Tabriz) as well as in other major cities in Iran follow strict national and WHO’s recommended standards; so the doubt about treated water quality distributed by public network is related to lack of awareness between participants. A research conducted in 2014 by McLeod et al in rural areas of Saskatchewan (Canada) showed that 25% of consumers were not satisfied with the taste, odor and turbidity of tap water.^20^ Another study in Canada revealed that 33% and 31% of consumers were unsatisfied with the taste and odor of drinking water, respectively.^21^ In separate surveys on consumers’ reasons for choosing bottled water in the US, Canada and France, the concern about contamination of tap water was reported as 47%, 25% and 23%, respectively. In these studies, about 7%, 71% and 47% consumers mentioned the taste and odor problem as the main reason for choosing bottled water for drinking.^2^ Also, in numerous other studies on the logic behind tendencies towards bottled drinking water usage in other cities and courtiers, doubt about the quality of the treated tap water as well as taste and odor has been reported as major reasons of consumers.^3,22-28^

Sub-class analysis of the study findings indicated that only 6.1% of the highly educated bottled drinking water users confirm acceptability of the tap water quality in their region, while 51.4% of the consumers with lower/no academic education considered tap water as acceptable for drinking purposes. The difference observed between the two groups was statistically significant (*P*=0.018). Other findings of the study revealed that keeping empty PET bottles for re-use for instance as a container for food stuff such as vinegar or lemon juice is more common amongst the study participants with lower educational attainments. About 60% of the study respondents (59.2%) reported segregation of empty PET bottles from other domestic wastes at the source of generation for recycling. Definitely, due to mismanagement and shortcomings in municipality facilities, all of the separated empty bottles by consumers are not supposed to be recycled. It is estimated that only about 10% of recyclable items in solid waste streams in Iran is recycled.^29^

Our findings are in good agreement with Zhang and Wen’s who reported that 44% of consumers segregated (polyethylene terephthalate) PET bottles, while 56% discarded them along with other wastes.^30^ Accordingly, more than half of the consumers adhered to segregation of PET bottles which can play an important role in recycling and final disposal of empty bottles if implemented. According to the results presented Table S1, about 90.8% of the participants believed that empty bottles may cause environmental problems when discarded into the nature. It has been reported that worldwide less than one-fifth of bottle wastes are disposed properly and most of them are dumped to seas, oceans, and landfills.^1^ For 71.7% of the participants, it was convenient to purchase bottled water and was affordable for them, while it was a financial burden for 27.5% of the respondents. So, the consumers might not spend money for buying bottled water if they are informed and ensured about tap water quality. Additionally, the results showed that about 46.5% of the consumers promoted using bottled water and suggested bottled water consumption to others. In this study, the consumers who suggested bottled water consumption to others had higher income, and did not consider dumping empty bottles as an environmental problem. According to a study conducted in Beijing (China), age, occupation and gender variables were important factors with regards to using drinks in PET bottles, while income was not an important factor.^30^ It has been found that in Canada, age, income, and academic degrees influence individuals’ perception and may lead them toward using bottled water. For instance, it has been reported that younger participants with lower income and academic degrees as well as older participants with higher income and academic degrees were more satisfied with tap water.^21^

According to Table 1, the consumption rate of bottled water has increased with population growth and due to increased demand. Nonetheless, it did not increase considerably in the last decades of the 20^th^ century in the country; but, in the early years of the new century, the rate started to rise dramatically especially after 2002. Between 2000 and 2015, on average about 1.04 billion liters of bottled water was consumed annually in Iran, where the annual per capita consumption rate of the bottled drinking water use increased from 0.41 liters in 2000 to 48.9 liters in 2015 (implying about 119-fold increase in bottled water consumption).

Based on the consumption trend of bottled water, the total estimated amount of PET bottle wastes grew from 822.58 tons in 2000 to 120,188 tons in 2015, representing an average annual production of 32,387 tons of PET bottle wastes between 2000 and 2015 (production of 424.17 g of PET wastes per capita). The per capita generation rate of the PET bottle wastes indicated a sharp increase from 12.84 g to 1519.38 g within a 15-year time period (equal to about 118-fold increase). Furthermore, the average per capita rate in this period was 424.17 g.

As presented in Figure 1, in 2000, bottled water consumption rate in Iran was 40 times lower than the world average rates, but in 2012, the difference decreased to only 1.5 times. On the other hand, in 2013, the trend was reversed such that the per capita bottled drinking water use in Iran overtook the world average from 2013 to 2015. The swift increase in bottled water consumption in Iran can be attributed to growing concerns about tap water quality, assuming that bottled water has better quality than tap water, little understanding about mineral water and bottled water, changes in life style, and welfare improvements in a group of people, etc.

According to the results presented in Figure 2, it can be seen that, the bottled water consumption in Iran is lower than some countries such as Germany, France, Spain, and the world average, but higher than some others including Denmark, Norway, and the Netherlands. Some of the selected countries in this figure have a public water supply system with treated potable water, but some other countries do not provide such a distribution system. It seems that bottled drinking water use in countries with public treated water supply might be influenced by other contextual factors such as lifestyle, economic conditions, and social beliefs, etc.

It should be reemphasized that the increasing rate of bottled water consumption in countries such as Iran which provides public drinking water network potable water supply system is very important in terms of economy, resource conservation, and management. Without recycling, all the waste generated from bottled water must be sent to landfill sites, released in the environment or transferred to seas and oceans. Then, due to non-biodegradability, low compaction potential, and high volume, these kinds of wastes could be a serious health and environmental challenge. If the plastic is incinerated or burned in open dumpsites, it creates hazardous air pollutants. On the other hand, supplying clean water by the public water supply system given Iran’s very limited water sources, with people in some cities showing a tendency to use bottled water, is another subject that authorities and experts should take into account.

The trend of waste bottle generation between 2000 and 2028 is displayed in Figure 3. As presented, the per capita PET bottled water waste production in Iran from 2000 to 2006, has been almost constant the growth trend, and from 2006 to 2015, there has been a significant increase. Also, the forecast of the per capita PET bottled water waste production, it will increase dramatically after 2015, and it will continue until 2021. The increment rate after 2021 will be far lower compared to the previous years. Until 2028, the upward trend will be gentle, and it is going to reach a staggering level of about 2500 g in 2028.

In Iran, 120 188 tons of discarded bottle waste were produced only in 2015. When considering other types of plastic as well, the amount of plastic waste would be huge. Waste management legislation in Iran was proposed by the government and approved by the parliament in 2004. According to Clause 12 of the executive instruction included in the legislation, all producers and importers of polymeric materials including plastics and rubber should recycle their waste; otherwise they must pay 0.005% of the value of produced**/**imported goods to a special fund, which should be used for recycling of the waste. As cited in the notes of Clause 12 of the executive instruction, companies that do not accept their responsibility must pay financial penalties. In contrast, companies that use recycled materials in their production processes are granted an exemption along with manufacturers/importers that voluntarily receive products back at the end of their useful lifespan or export their products. The Ministry of Health and National Environmental Protection Agency is responsible for proper implementation of the legislation.^31,32^ However, this legislation has not been implemented properly and completely for recycling PET and bottled water wastes.

In the studied area, people did not separate all of generated plastic wastes, especially bottled water wastes. Those citizens who segregated wastes at the source could give their segregated recyclable waste such as PET bottles for recycling in two methods:

1. Buy-back centers: Segregated wastes delivered to recycling stations located in different areas of the city.
2. Drop-off centers: Segregated wastes dumped into special boxes located in some regions and streets of the city.

These methods have recently been used for collecting PET bottles and other recyclable wastes. However, the methods are useless since most of collected wastes are not segregated, and are mixed with other municipal wastes, which are finally landfilled instead of proper recycling. In Tabriz’s landfill site, there are both a material recycling facility and illegal recyclers who separate PET bottles in a completely unsanitary condition. Most probably, the situation is the same in most of the other cities in the country.

Numerous studies have been conducted on the waste management of PET bottles worldwide. Some of the introduced methods might be more efficient in a number of countries than in others. Yet, it is important to note that the existing infrastructure could play a pivotal role in reaching success and therefore, different suggested methods may lead to diverse outcomes in various countries of the world.

China is the largest PET bottle consumer in the world and the production of PET bottles in this country has increased by more than 12-fold, specifically from 0.25 million tons to 3.2 million tons during 2003-2010.^30^ China is followed by the United States; in which the PET bottle recycling rate decreased from 40% to 29% between 1995 and 2010.^33^ The National Association for PET Container Resources (NAPCOR) as the responsible body for the PET bottle recycling was established in the US in 1987 which provides annual reports about its activities.^34^ According to the NAPCOR report, approximately 55% of the PET containers collected for recycling are generated through curbside programs.^34^ Based on the results of other studies in the US, nearly 70%-90% of recyclable wastes could be managed by the curbside method.

Other common methods for collecting and recycling PET bottles in the US included:

Deposit-refund scheme: the deposit return system based on the *Container*Deposit* Legislation* (CDL, enacted in 1982) endorses receiving a refundable deposit from end-line users of recyclable beverage containers to ensure their recycling.

The curbside collection method: this method is one of the most convenient and efficient methods for collecting empty PET bottles, encouraging active cooperation of the community members.

Establishing drop-off centers: in this method, community members deliver their recyclable PET wastes to certain collection centers. This method has a lower implementation cost compared to the curbside method, but is less preferable for citizens due to inconvenience of collecting and transporting the collected wastes to the drop-off sites. Nevertheless, establishing drop-off centers in remote and rural areas could be a cost-efficient solution, where application of other methods such as curbside collection is not feasible.

Establishing buy-back centers: in this method, pre-arranged collection sites buy empty PET bottles from consumers. In most cases, private sectors run such collection repositories.^34,35^

In Europe, about 1.8 million tons of PET bottles were collected in 2014 for recycling purposes, which is about 5% greater than the respective amount of the previous year.^36^ In Germany, according to the results of a study in 2016, about 97% of PET bottles were collected and almost 80% were recycled (30% bottle-to-bottle recycling and 70% bottle-to-fiber recycling). According to the information in a published report, 90% of PET bottles were collected, with the recycling rate being 85.5% in Sweden. In the Netherlands, based on a compulsory law in this country, soda and bottled water producers have to incorporate recycled PET in their manufacturing process.^37^ In Lithuania, about 15,139 tons of PET bottles were produced in 2014^37^ and 10,239 tons of which were dumped in the municipal waste sites, with only 4 900 tons collected separately. In Japan, the extended producer responsibility (EPR) strategy is used for PET bottle management in order to promote incorporating whole lifetime environmental costs of goods production into the market prices. Japan as with Germany has the highest PET recycling rate among the developed countries. It managed to increase the recycling rate of PET bottles by 70% during 1995-2010 through implementing the EPR strategy. The Council for PET Bottle Recycling in close collaboration with the Containers and Packaging Recycling Association in Japan are the responsible bodies for collecting and recycling of empty PET bottles, which provide annual reports about their achievements in PET bottle recycling.^38^

## Conclusion

Considerable perceived uncertainty was observed about the quality of the treated tap water in this study. On average, 74% of participants considered tap water unacceptable from different aspects of quality. Participants with higher academic education did not consider tap water as a safe source for drinking purposes. In response, growing tendencies towards bottled drinking water use was evident amongst the study participants and the whole country. Bottled water consumption has increased from 0.41 to 48.9 L/per capita-year between 2000 and 2015. Considering the volume of PET bottled water wastes generated in Iran (120187.95 tones) during 2015 and since the potable drinking water meets national and international standards in most urban and even rural areas of the country, prompt intervention is recommended to ban the bottled drinking water usage rise at the first stance. Also, a national plan should be prepared and implemented for the PET waste management. As a starting point, educational programs for raising public awareness about the quality of tap water, policy changes to limit PET bottled drinking water use (e.g. taxation procedure), and establishing a national system (such as EPR) for better management of PET wastes are highly recommended.

## Acknowledgments

The authors wish to thank all members of the research team and others who participated in this study.

## Funding

This work was funded by Research Deputy of Tabriz University of Medical Sciences.

## Competing interests

The authors declare that they have no conflict of interest.

## Ethical approval

Not applicable for this study.

## Authors’ contributions

All authors contributed to the study conception and design. Material preparation, data collection, and analysis were performed by HA, PP, AM, AS, HT, and MZ. The first draft of the manuscript was written by MZ, and all authors commented on previous versions of the manuscript. All authors read and approved the final manuscript.

**Table 1:** Amounts of consumed bottled drinking water and associated PET wastes produced in Iran

**Year**	**Consumed bottled water (tons)**	**Consumed bottled water** **(10** ^ 9 ^ ** L)**	**Number of the used 0.5 L bottles (10** ^ 6 ^ **)**	**Number of the used 1.5 L bottles (10** ^ 6 ^ **)**	**PET Wastes associated with bottled water use (tons)**	**Population (10** ^ 6 ^ **)**	**Bottled drinking water consumption** **(L/ capita-year)**	**Produced PET bottle Wastes** **(g/capita-year)**
1962	1768	0.0017	2.12	0.47	54.93	22.80	0.07	2.40
1975	2668	0.0026	3.20	0.71	82.89	32.81	0.08	2.52
1987	11678	0.011	14.01	3.11	362.8	50.68	0.23	7.15
2000	26478	0.026	31.77	7.06	822.58	64.04	0.41	12.84
2001	27,278	0.027	32.73	7.27	847.44	65.08	0.42	13.02
2002	29,918	0.029	35.90	7.98	929.45	66.13	0.45	14.05
2003	60,958	0.060	73.15	16.26	1893.76	67.21	0.91	28.18
2004	83,198	0.083	99.84	22.19	2584.68	68.29	1.22	37.85
2005	156,020	0.156	187.22	41.61	4847.03	69.35	2.25	69.89
2006	102,181	0.102	122.62	27.25	3174.43	70.50	1.45	45.03
2007	294,039	0.294	352.85	78.41	9134.82	71.13	4.13	128.43
2008	648,532	0.648	778.24	172.94	20147.71	71.99	9.01	279.85
2009	981,930	0.981	1178.32	261.85	30505.31	72.87	13.47	418.6
2010	1,242,212	1.242	1490.65	331.26	38591.37	74.20	16.74	520.07
2011	1,544,171	1.544	1853.01	411.78	47972.24	75.15	20.55	638.36
2012	1,827,657	1.827	2193.19	487.38	56779.21	76.12	24.01	745.93
2013	2,560,966	2.560	3073.16	682.92	79560.68	77.10	32.22	1031.9
2014	3,226,135	3.226	3871.36	860.30	100225.27	78.10	41.33	1283.37
2015	3,868,711	3.868	4642.45	1031.66	120187.95	79.10	48.91	1519.38

**Appendix:**